# Ni(OH)_2_ Decorated Pt-Cu Octahedra for Ethanol Electrooxidation Reaction

**DOI:** 10.3389/fchem.2019.00608

**Published:** 2019-09-03

**Authors:** Youngmin Hong, Hee Jin Kim, Hye Jin Lee, Jeonghyeon Kim, Sang-Il Choi

**Affiliations:** Department of Chemistry and Green-Nano Materials Research Center, Kyungpook National University, Daegu, South Korea

**Keywords:** copper, platinum, nickel hydroxide, octahedra, ethanol oxidation

## Abstract

Here we report the synthesis of 9 nm Ni(OH)_2_ decorated Pt-Cu octahedra (Ni(OH)_2_-PtCu) in one-pot synthesis for ethanol oxidation reaction (EOR) electrocatalysis in acidic electrolyte. To prepare Ni(OH)_2_-PtCu octahedra, CO gas was directly introduced in a reaction process as selective capping agents on the PtCu(111) facet. Ni(OH)_2_ was naturally deposited on the Pt-Cu octahedra during the synthesis. Carbon supported Ni(OH)_2_-PtCu (Ni(OH)_2_-PtCu/C) as an EOR catalyst showed enhanced CO tolerance due to the existence of oxophilic Ni(OH)_2_ on the surface of Pt-Cu, facilitating water dissolution to produce OH adsorption and to promote complete CO oxidation to CO_2_. In addition, Pt-Cu alloy composition also showed improvement of CO tolerance because of modified *d*-band structure of the Pt atoms, thereby weakening the binding strength of CO on the catalysts. Therefore, the Ni(OH)_2_-PtCu/C showed enhanced EOR activity and durability compared to the Pt-Cu octahedra and commercial Pt/C counterparts.

## Introduction

Direct alcohol fuel cell (DAFC) for numerous applications has attracted huge attention due to its high efficiency under ambient operating conditions (Xu and Zhang, 2014; Ozoemena, 2016). For the anodic reaction of DAFC, ethanol has been received great interests for its utilization owing to its high energy density (8 kWh kg^−1^), low toxicity, and ease of supply from agricultural products and biomass (Li and Pickup, 2006; Xu and Zhang, 2014). In addition, ethanol can be easily stored and transported for the practical uses compared to H_2_ gas (Zhou et al., 2003; Kowal et al., 2009). During the ethanol oxidation reaction (EOR) in fuel cells, ethanol is consumed to generate electrical energy, and CO_2_ is generated as a product from complete oxidation of ethanol. In this process, 12 electrons are produced (CH_3_CH_2_OH + 3H_2_O → 2CO_2_ + 12H^+^ + 12e^−^) under the thermodynamic standard potential of 0.085 V (Colmati et al., 2009; Busó-Rogero et al., 2014). However, owing to the slow kinetics of the EOR, making large overpotential and concomitant intermediate such as CO that has toxicity to the environment and fuel cell electrode materials, a number of researches have been made on new catalysts for enhancing EOR kinetics, CO-tolerant, and CO oxidation to CO_2_ (Neto et al., 2007; Busó-Rogero et al., 2014; Ozoemena, 2016).

Platinum (Pt) has been commonly used as an EOR catalyst due to its excellent properties in the adsorption and dissociation of ethanol (Wang et al., 2015). However, high-cost, low abundance in the Earth crust, and undesirable CO poisoning during the EOR have delayed the practical applications (Zhang et al., 2015; Ghavidel et al., 2017). Therefore, alloying Pt with transition metals, such as Ru, Sn, Pd, Ni, and Cu have been intensively studied to reduce the Pt use and to alleviate CO poisoning on the surface of Pt thus to enhance the electrocatalytic activity of EOR (Vigier et al., 2004; Colmenares et al., 2006; Wang et al., 2006; Ammam and Easton, 2013; Dutta and Ouyang, 2015; Zhu et al., 2015). In this Pt-based alloy nature, a bifunctional mechanism was generally accepted to rationalize enhanced performance of EOR. Specifically, the transition metals (M) positioned near the surface Pt would dissociate the water molecule and act as an adsorbent for the produced OH on its surface site (H_2_O → M-OH_ads_ + H^+^ + e^−^) at lower potentials compared to Pt (Hsieh and Lin, 2009; Kowal et al., 2009). Then, the M-OH_ads_ on the surface of catalyst can help the oxidation of CO to CO_2_ on the neighboring Pt sites (Pt-CO_ads_ + M-OH_ads_ → CO_2_ + H^+^ + e^−^) (Koper, 2011; Erini et al., 2015). Moreover, the M alloyed into the lattice of Pt can modify electronic structure of Pt, resulting in the reduction of bonding strength of Pt and CO and thus increase of EOR performance (Zhang et al., 2016; Liu et al., 2018). Additionally, ternary nanostructured catalysts were also studied to derive ternary ensemble of each metals. For example, Pt-Rh-Sn and Pt-Rh-Ni ternary alloys possessing highly oxophilic Rh, Sn, and Ni elements have been introduced to further modify the property of Pt-based bimetallic catalysts (Erini et al., 2015). These results indicated that the surface modification of alloy catalyst with oxidation form of transition metals such as Ni(OH)_x_ activates water dissociation and provides OH_ads_ at lower potentials than Pt (Cui et al., 2013a; Erini et al., 2015).

Recently, facet-dependent electrocatalytic EOR has been intensively studied with the nanocatalysts of unique morphologies (Lai and Koper, 2008; Koper, 2011; Solla-Gullon et al., 2011; Zhao et al., 2016). In the case of Pt-Ni alloy, octahedral Pt-Ni nanocatalysts enclosed by (111) facets showed 4.6 times higher EOR activity compared to spherical Pt-Ni consisted of mixed facets (Sulaiman et al., 2017). To the best of our knowledge, however, there has not much reports on ternary Ni(OH)_2_-Pt-Cu electrocatalyst for EOR. In this context, we here successfully synthesized 9 nm Ni(OH)_2_ decorated Pt-Cu octahedra in one-pot synthesis. We designed Pt-Cu octahedra as PtCu(111) alloy substrates and Ni(OH)_2_ decorated on the surface of Pt-Cu octahedra as the site for OH_ads_, improving water dissociation. The as-obtained Ni(OH)_2_ decorated Pt-Cu octahedra showed enhanced catalytic EOR activity and durability compared to binary Pt-Cu octahedra and commercial Pt/C.

## Experimental Section

### Chemicals

Platinum(II) acetylacetonate [Pt(acac)_2_, Pt 48.0%] and copper(II) acetylacetonate [Cu(acac)_2_, 98%] were obtained from Alfa Aesar. Nickel(II) acetylacetonate [Ni(acac)_2_, 95%], oleic acid (OAc, 90%), oleylamine (OAm, ≥98%), and benzyl ether (BE, 98%) were obtained from Sigma-Aldrich. All chemicals were used as received without further treatment.

### Synthesis of Ni(OH)_2_ Decorated Pt-Cu Octahedra (Ni(OH)_2_-PtCu)

In a standard synthesis, a mixture of Pt(acac)_2_ (20.0 mg), Cu(acac)_2_ (13.3 mg), Ni(acac)_2_ (10.0 mg), OAm (4.0 mL), OA (4.0 mL), and BE (2.0 mL) was heated to 190°C within 20 min under Ar atmosphere and magnetic stirring. When the temperature reached 190°C, CO gas was bubbled into the mixture with a flow rate of 1 mL min^−1^, and Ar purging was stopped at the same time (Kang et al., 2010). The mixture was then heated to 210°C at a heating rate of 4°C min^−1^ under CO bubbling and held at 210°C for 40 min without CO bubbling. The resulting suspension was cooled down to room temperature naturally, and the Ni(OH)_2_-PtCu octahedra were precipitated out by sequential addition of toluene (5 mL) and ethanol (10 mL). The supernatant was discarded by centrifugation at 3,000 rpm for 5 min. The resulting Ni(OH)_2_-PtCu octahedra were dispersed in toluene for further treatment.

### Preparation of Ni(OH)_2_-PtCu Octahedra Supported on Carbon (Ni(OH)_2_-PtCu/C)

A suspension of Ni(OH)_2_-PtCu octahedra was added into a toluene solution containing 31.5 mg of Vulcan XC-72 R carbon and kept under ultrasonic wave agitation for 2 h. The resulting Ni(OH)_2_-PtCu/C catalyst was centrifuged 3 times with toluene at 3,000 rpm for 5 min, and then dried under Ar protection at room temperature.

### Acetic Acid (HOAc) Treatment of Ni(OH)_2_-PtCu/C Catalyst (PtCu/C-HOAc)

Ni(OH)_2_-PtCu/C catalyst (10 mg) was dispersed in HOAc (10 mL; ≥99.7%, Sigma-Aldrich) and then heated at 60°C for 2 h under magnetic stirring. After heating, the catalyst was washed three times with ethanol and dried under Ar protection at room temperature.

### Morphological, Structural, and Elemental Characterizations

Transmission electron microscopy (TEM) images were obtained using a HT 7100 microscope (Hitachi, Japan) operated at an acceleration voltage of 120 kV. High-resolution TEM and energy dispersive X-ray spectroscopy (EDS) studies were carried out in JEM-2100F (JEOL, Japan) and Titan G2 ChemiSTEM Cs Probe (FEI, USA) operated at an acceleration voltage of 200 kV. The metal contents in catalysts were determined using inductively coupled plasma-optical emission spectroscopy (ICP-OES, PerkinElmer, Optima 7300DV, USA). X-ray diffraction (XRD) patterns were obtained with a D2 phaser X-ray diffractometer (Bruker, USA). X-ray photoelectron spectroscopy (XPS) was carried out using a spectrometer (Thermofisher Scientific, USA) with Al Kα X-ray (1486.6 eV) as the light source. All spectra were aligned using the C-1s peak at 284.5 eV as reference.

### Electrochemical Measurements

All electrochemical measurements were carried out using a standard three-electrode cell connected to potentiostats (CHI 600E from CH Instruments and VSP from Bio-Logic). A Ag/AgCl electrode was used as the reference electrode, and potential values were calibrated with respect to reversible hydrogen electrode (RHE). Pt mesh (1 × 1 cm^2^) was used as a counter electrode. A glassy carbon disk (GC, 5 mm in diameter, PINE instrumentation) was used as a working electrode. The GC surface was previously polished using 0.05 μm Al_2_O_3_ suspension. Catalyst ink was prepared by mixing a specific amount of catalyst powder, deionized water (1.0 mL), isopropyl alcohol (0.25 mL), and 5 wt% nafion (5 μL) using ultrasonication for 10 min. The ink (10 μL) was dropped on a GC electrode and dried to form a thin film. The final Pt loading of electrocatalyst on a GC electrode was 10.2 μg cm^−2^. The each electrocatalyst loaded on electrode was pre-cycled in an Ar-saturated 0.1 M HClO_4_ solution between 0.08 and 1.20 V at a scan rate of 100 mV s^−1^, and then cyclic voltammograms (CVs) were recorded between 0.08 and 1.20 V at a scan rate of 50 mV s^−1^ in an Ar-saturated 0.1 M HClO_4_ solution at room temperature. In a typical CO stripping experiment, the electrode potential was held at 0.05 V for 5 min in a CO-saturated 0.1 M HClO_4_ solution for a fully adsorption of CO on the catalyst surface followed by flowing Ar for another 10 min to remove the CO in the solution, and then the CO stripping curve was recorded by cycling between 0.08 and 1.20 V at 50 mV s^−1^. For the EOR measurements, CV was conducted in an Ar-saturated solution containing 0.1 M HClO_4_ and 1 M ethanol between 0.08 and 1.29 V at a scan rate of 50 mV s^−1^. Chrono-amperometry curve of the catalysts was recorded for 3000 s in an Ar-saturated 0.1 M HClO_4_ and 1 M ethanol solution at 0.67 V. Long-term durability of the catalysts for EOR was performed in an Ar-saturated 0.1 M HClO_4_ and 1 M ethanol solution by applying cyclic potential sweep between 0.65 and 1.05 V at a scan rate of 100 mV s^−1^ for 2,000 cycles. Then, CV for EOR was recorded again in a fresh Ar-saturated 0.1 M HClO_4_ and 1 M ethanol solution for comparison with the initial CV curve of EOR.

## Results and Discussion

**Figure 1:**
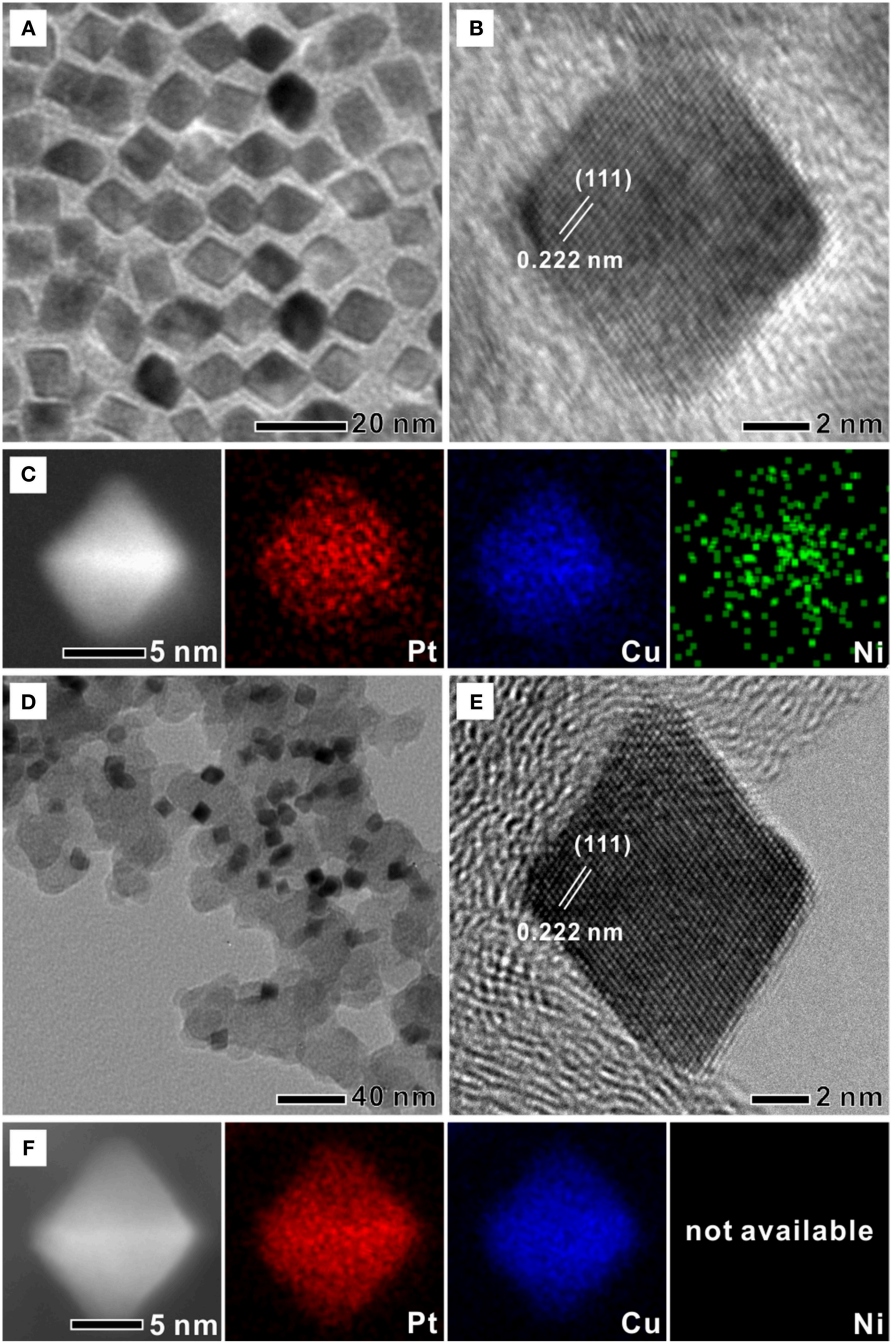
TEM and high-resolution TEM images of **(A,B)** Ni(OH)_2_ decorated Pt-Cu octahedra (Ni(OH)_2_-PtCu) and **(D,E)** Ni(OH)_2_ decorated Pt-Cu octahedra/C treated with acetic acid (PtCu/C-HOAc). STEM with corresponding EDS mapping images of **(C)** Ni(OH)_2_-PtCu and **(F)** PtCu/C-HOAc.

In a typical synthesis of Ni(OH)_2_-PtCu octahedra, a reaction mixture of Ni(acac)_2_, Pt(acac)_2_, Cu(acac)_2_, OAc, OAm, and BE was heated to 190°C under Ar protection with magnetic stirring and then CO gas was bubbled into heated mixture with a flow rate of 1 mL min^−1^ while Ar purging was stopped. The mixture was heated again to 210°C, and CO bubbling was stopped when the temperature reached to 210°C. After the reaction was kept at 210°C for 40 min, a dark suspension including Ni(OH)_2_-PtCu was obtained. In previous studies, CO gas released from metal carbonyls was used as a selective capping agent for {111} facets of Pt-Ni and Pt-Cu alloy nanocrystals under an OAm/OA co-surfactant system (Choi et al., 2013). However, use of transition metal carbonyl compounds can cause undesirable trace metal contaminations on the Pt catalysts through alloying and/or surface residues (Kang et al., 2010, 2013). Furthermore, transition metals released from metal carbonyls complicate the synthesis system, making it difficult to identify the formation mechanism of shape-controlled nanocrystals (Chang et al., 2017). Therefore, we here simply used CO gas as selective capping agent directly into the reaction mixture. Figure 1A shows TEM image of Ni(OH)_2_-PtCu octahedra with an average edge length of 9.4 ± 1.1 nm. A TEM image on a large population of Ni(OH)_2_-PtCu octahedra shows uniform size and shape (Figure S1A). High-resolution TEM (HRTEM) image of a single Ni(OH)_2_-PtCu octahedron shows that the *d*-spacing for adjacent Pt lattice fringes measured from a few different sites was 0.222 nm, which is smaller than that of Pt{111} planes (0.227 nm) of face-centered cubic (fcc) bulk (Figure 1B) (Choi et al., 2013). Scanning transmission electron microscopy (STEM) with energy dispersive X-ray spectroscopy (EDS) mapping image confirmed well distributed Ni, Pt, and Cu elements in the single Ni(OH)_2_-PtCu octahedron (Figure 1C). The Ni(OH)_2_-PtCu octahedra loaded on carbon supports (Ni(OH)_2_-PtCu/C) were shown in Figure S1B, indicating well-dispersed nanocrystals on carbon without aggregation. The Ni(OH)_2_-PtCu/C was then treated with HOAc at 60°C for 2 h. Figures 1D,E show TEM and HRTEM images of Ni(OH)_2_-PtCu/C after HOAc treatment (PtCu/C-HOAc), respectively. After the acid treatment, the average edge length of PtCu/C-HOAc was 9.2 ± 1.1 nm and *d*-spacing for Pt lattice fringes was 0.222 nm, similar to the pristine Ni(OH)_2_-PtCu octahedra. STEM with EDS mapping image of a single PtCu/C-HOAc shows an octahedral nanocrystal consisting of Pt and Cu but absence of Ni (Figure 1F).

To confirm the elemental composition of the as-prepared catalysts, ICP-AES was carried out and the resulting data are listed in Table 1. The ICP-AES results of Ni(OH)_2_-PtCu/C and PtCu/C-HOAc indicate that Ni was fully removed and Cu was partially leached out during the HOAc treatment. Previous literatures on the Pt-based alloys have found that the transition metals in the Pt lattices cannot be completely removed during the acid treatment, but some are retained in the crystal structure (Chang et al., 2017; Park et al., 2017; Kwon et al., 2018; Kim et al., 2019). Therefore, in our case, we suggest that Ni was selectively deposited on the surface of Pt-Cu octahedra during the synthesis and was dissolved out when the Ni(OH)_2_-PtCu/C catalyst was treated with HOAc. The Cu atoms on the surface of Ni(OH)_2_-PtCu/C were also leached out and thus the surface composition of PtCu/C-HOAc is assumed to be Pt-rich.

**Table 1:** Comparison of atomic ratio and Pt wt% in a catalyst obtained by ICP-AES, H_upd_-based, and CO_ads_-based electrochemical surface areas (ECSA_H_, ECSA_CO_), and onset potential of CO stripping.

**Catalyst**	**Atomic ratio** **Ni: Pt: Cu**	**Pt wt% in a catalyst**	**ECSA_H_** **(m^**2**^${\text{g}}_{\text{Pt}}^{-1}$)**	**ECSA_CO_** **(m^**2**^${\text{g}}_{\text{Pt}}^{-1}$)**	**Onset potential of CO (V)**
Commercial Pt/C	NA^*^	20^**^	60.29	66.83	0.80
Ni(OH)_2_-PtCu/C	1.0: 2.1: 2.8	14.1	27.27	27.34	0.63
PtCu/C-HOAc	NA: 1.0: 1.0	14.9	28.06	30.85	0.67

* *Not applicable*.

** *Nominal amount written in a tag*.

**Figure 2:**
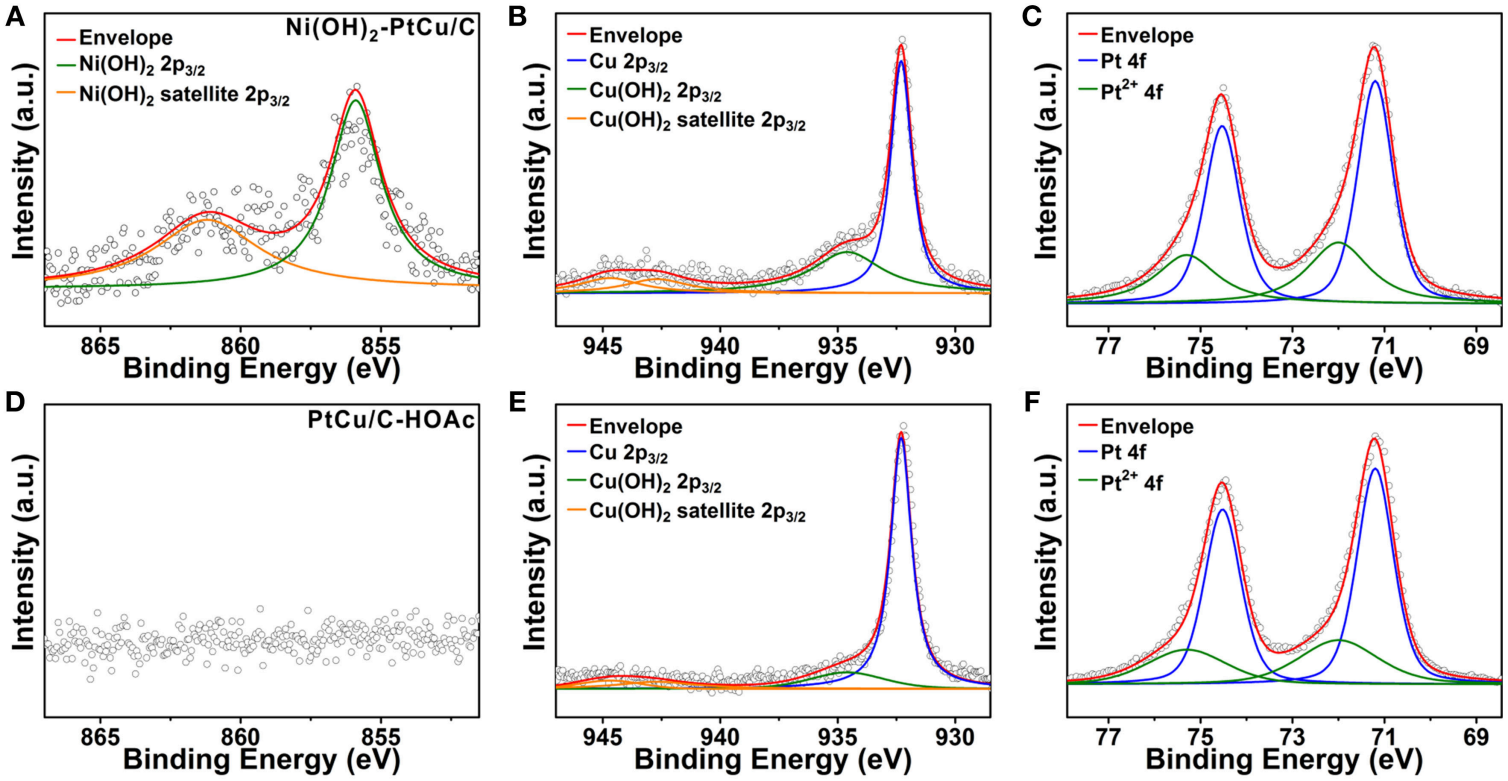
Ni 2p_3/2_ XPS spectra of **(A)** the Ni(OH)_2_-PtCu/C and **(D)** the PtCu/C-HOAc. Cu 2p_3/2_ XPS spectra of **(B)** the Ni(OH)_2_-PtCu/C and **(E)** the PtCu/C-HOAc. Pt 4f XPS spectra of **(C)** the Ni(OH)_2_-PtCu/C and **(F)** the PtCu/C-HOAc.

**Figure 3:**
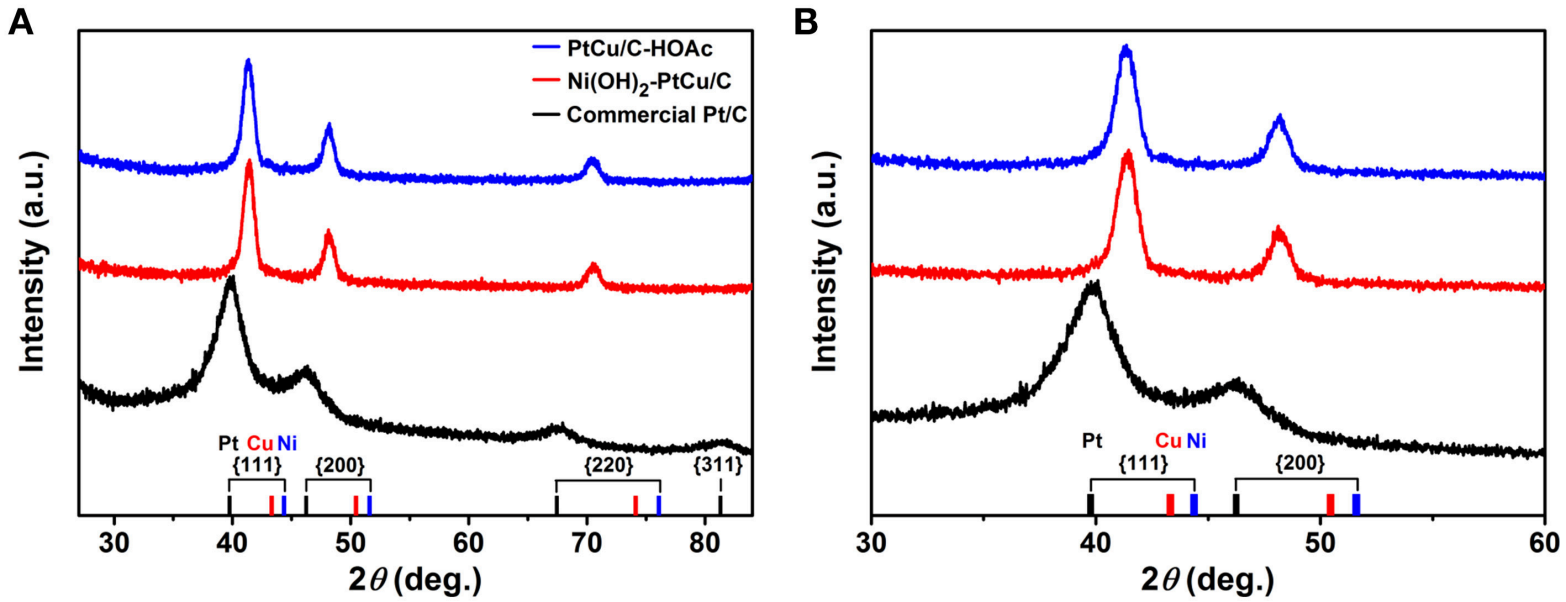
XRD patterns of Ni(OH)_2_-PtCu/C, PtCu/C-HOAc, and commercial Pt/C in **(A)** wide and **(B)** narrow 2θ ranges.

To investigate chemical state of elements in catalysts, XPS was conducted. The Ni 2p_3/2_ spectrum of Ni(OH)_2_-PtCu/C shows a peak assigned to Ni(OH)_2_ (855.9 eV) and Ni(OH)_2_ satellite (861.2 eV), indicating the formation of Ni(OH)_2_ on the surface of Ni(OH)_2_-PtCu/C (Figure 2A) (Moulder, 1992). Meanwhile, Ni signal was not detected for PtCu/C-HOAc, revealing again that surface Ni(OH)_2_ was dissolved when catalyst was treated with HOAc (Figure 2D). The Cu 2p_3/2_ spectra of both Ni(OH)_2_-PtCu/C and PtCu/C-HOAc showed a peak assigned to Cu(0) metal (932.3 eV), Cu(OH)_2_ (934.6 eV), and Cu(OH)_2_ satellite (942.7 and 944.7 eV) (Figures 2B,E) (Moulder, 1992). The Cu(OH)_2_ peak was decreased after the HOAc treatment, indicating the surface Cu(OH)_2_ leaching similar to Ni(OH)_2_. The Pt 4f spectra of both catalysts show a peak fitted with doublets corresponding to Pt(0) metal (71.2 and 74.5 eV) and Pt^2+^ (72.0 and 75.3 eV) (Figures 2C,F) (Xia et al., 2012). Figure 3A shows XRD patterns in a wide 2θ range for commercial Pt/C, Ni(OH)_2_-PtCu/C before and after HOAc treatment, representing fcc structure without other metal oxide peaks. The narrow 2θ range window of the XRD shows diffraction peaks between Pt and Cu (Figure 3B), indicating that the decreased lattice spacing of Pt for Ni(OH)_2_-PtCu/C and PtCu/C-HOAc compared to commercial Pt/C is the result of the presence of smaller Cu atoms in place of Pt atoms (Nosheen et al., 2013). Based on the observations, we understand the formation of Ni(OH)_2_-PtCu octahedra during the standard synthesis. In the synthesis, Pt^2+^, Cu^2+^, and Ni^2+^ ions were initially decomposed out from the introduced metal precursors. Because of the difference of standard reduction potentials, Pt^2+^ (1.188 V) and Cu^2+^ ions (0.340 V) were primarily reduced to Pt and Cu metals forming Pt-Cu alloy nanocrystals but Ni^2+^ (−0.257 V) was dissolved in the mixture. Under the CO gas, Pt-Cu octahedra enclosed by {111} facets were mainly produced, and then rest of Ni^2+^ was deposited on the surface of Pt-Cu as Ni(OH)_2_ form (Zhang et al., 2015; Kavian et al., 2016).

**Figure 4:**
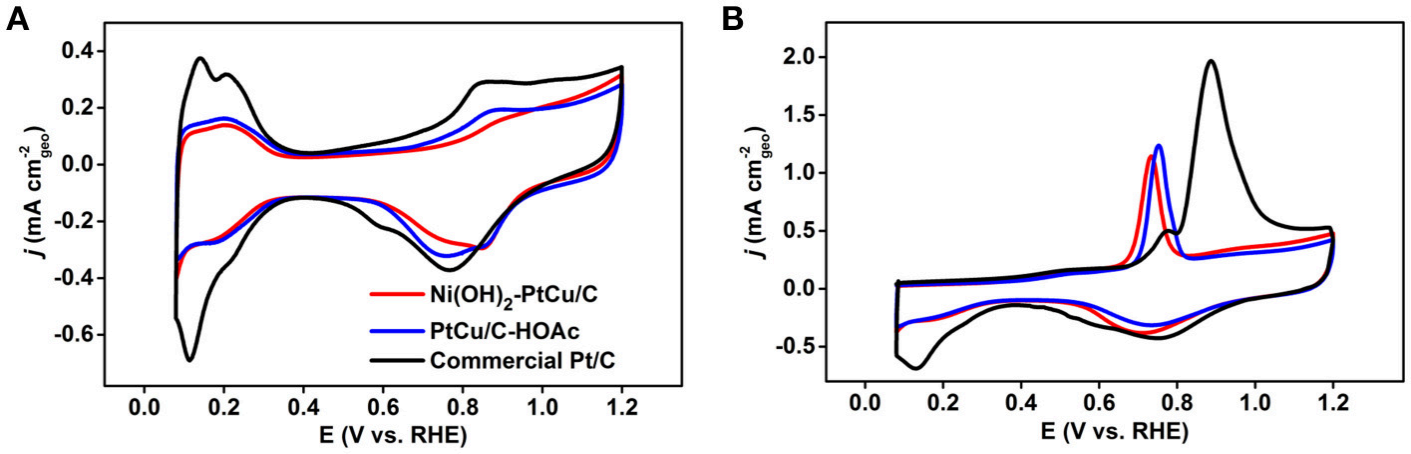
**(A)** Cyclic voltammograms (CVs) and **(B)** CO stripping curves of the Ni(OH)_2_-PtCu/C, the PtCu/C-HOAc, and the commercial Pt/C recorded in 0.1 M HClO_4_ solutions with a scan rate of 50 mV s^−1^ at room temperature.

**Figure 5:**
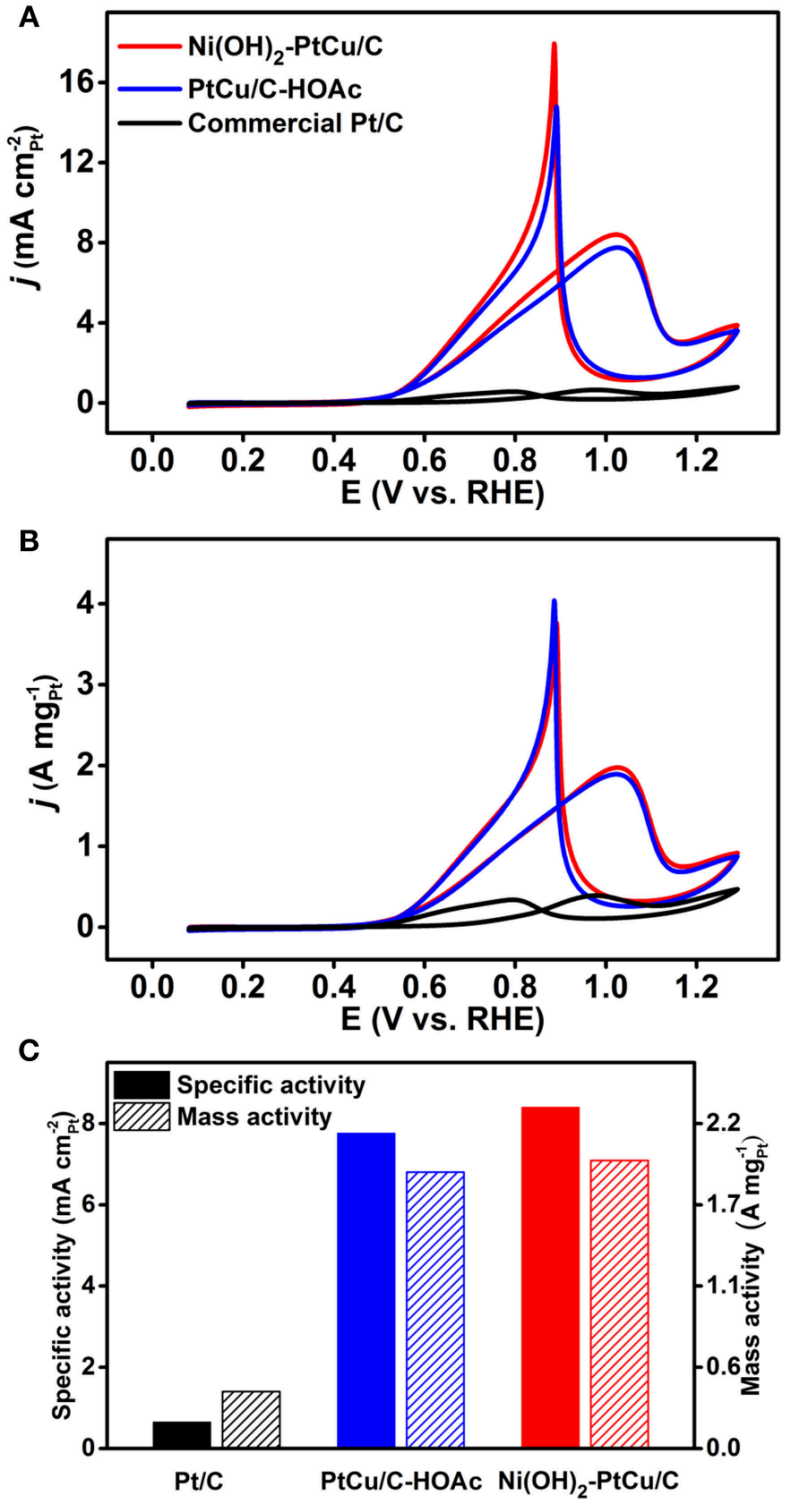
CVs of the Ni(OH)_2_-PtCu/C, the PtCu/C-HOAc, and the commercial Pt/C normalized by **(A)** ECSA and **(B)** Pt loading recorded in Ar-saturated solutions containing 0.1 M HClO_4_ and 1.0 M ethanol with a scan rate of 50 mV s^−1^ at room temperature. **(C)** Comparisons of the specific and mass activities toward the ethanol oxidation reaction of the three different catalysts measured at the potentials showing the maximum current density in forward scans.

The resulting Ni(OH)_2_-PtCu/C and PtCu/C-HOAc catalysts were evaluated as anodic oxidation electrocatalysts for EOR. For comparison, we benchmarked their electrochemical properties against the commercial Pt/C catalyst. Electrochemical measurements were conducted by using a potentiostats with three electrode system. The three different catalysts loaded working electrodes were pre-cleaned in Ar-saturated 0.1 M HClO_4_ solutions between 0.08 and 1.20 V (vs. RHE) with a scan rate of 100 mV s^−1^ at room temperature. Figure S2 presents TEM and EDS mapping images of Ni(OH)_2_-PtCu/C after cleaning, showing similar morphology and composition to its status before cleaning. CVs were then recorded with the same potential range at a scan rate of 50 mV s^−1^ as shown in Figure 4A. The electrochemically active surface area (ECSA) of Pt was estimated by measuring the charge associated with hydrogen adsorption/desorption region in CVs and those are 27.27, 28.06, 60.29 m^2^ g^−1^ for Ni(OH)_2_-PtCu/C, PtCu/C-HOAc, and the commercial Pt/C, respectively (Table 1, Rudi et al., 2014; Kim et al., 2017). The lower ECSA of Ni(OH)_2_-PtCu/C relative to that for the Pt/C is due to the bigger size and the presence of surface Ni(OH)_2_ (Park et al., 2017). ECSA based on hydrogen adsorption/desorption region in CV is mainly responsible for Pt surface of catalysts. Therefore, the slight increase in ECSA with PtCu/C-HOAc in comparison to that of Ni(OH)_2_-PtCu/C can be attributed to the removal of Ni(OH)_2_ from the catalyst surface after HOAc treatment (Kavian et al., 2016). A key limiting factor for EOR catalysts is the intermediate species (i.e., CO) generated during the ethanol oxidation (Huang et al., 2015). Thus, electrochemical CO stripping measurement was conducted to examine the CO oxidation on the all presented catalysts. Figure 4B shows the CO stripping curves of Ni(OH)_2_-PtCu/C, PtCu/C-HOAc, and the commercial Pt/C. The ECSAs were also calculated by CO stripping and those are 27.34, 30.85, 66.83 m^2^ g^−1^ for Ni(OH)_2_-PtCu/C, PtCu/C-HOAc, and the commercial Pt/C, respectively (Rudi et al., 2014; Kwon et al., 2018). The result is in accordance with the ECSA calculated by H adsorption/desorption. The onset potential for the CO stripping is usually used to prove the anti-CO poisoning property of the catalysts, in which lower potential values are related to higher CO tolerance (Zheng et al., 2015; Ahmad et al., 2019). The onset potential of the CO oxidation for Ni(OH)_2_-PtCu/C catalyst appeared at 0.63 V, exhibiting more negative shift than that on the PtCu/C-HOAc (0.67 V) and Pt/C (0.80 V) catalysts. This result imply weaker CO adsorption strength on the Ni(OH)_2_-PtCu/C and PtCu/C-HOAc catalysts than that on the Pt/C. (Xu et al., 2015; Sulaiman et al., 2017). As proposed by the *d*-band model, Pt alloying with Cu can induce lattice compression and thus down-shifting of the Pt *d*-band center, thereby weakening the CO adsorption energy (Yu et al., 2013; Hong et al., 2015; Zhang et al., 2015). In addition, since the CO stripping on Pt surface is sensitive to the surface arrangements, the octahedral shape of both the Ni(OH)_2_-PtCu/C and PtCu/C-HOAc has a narrower distribution of CO binding strength, resulting in the narrower CO oxidation peak as compared to spherical Pt/C enclosed by a mixed surface arrangement (Sulaiman et al., 2017). In the case of Ni(OH)_2_-PtCu/C, existence of Ni(OH)_2_ on Pt-Cu surfaces reduced the onset potential compared to the PtCu/C-HOAc owing to the presence of more OH adsorption sites to promote the CO oxidation, namely bifunctional effect (Erini et al., 2015; Xu et al., 2015). The CVs of the as-prepared catalysts for EOR were obtained in 0.1 M HClO_4_ and 1.0 M ethanol solutions between 0.08 and 1.29 V with a scan rate of 50 mV s^−1^ at room temperature. In order to evaluate the EOR activity, the currents were normalized with ECSA and mass of Pt loading, indicating the specific and mass activities as shown in Figures 5A,B, respectively. The specific activity of the Ni(OH)_2_-PtCu/C (8.40 mA cm^−2^) at the potential showing highest current density in a forward scan exceeded those of the PtCu/C-HOAc (7.76 mA cm^−2^) and the Pt/C (0.65 mA cm^−2^), respectively. The EOR mass activity of Ni(OH)_2_-PtCu/C is recorded the highest value of 1.97 A mg^−1^ compared to PtCu/C-HOAc (1.89 A mg^−1^) and the Pt/C (0.39 A mg^−1^) (Figure 5C). The enhanced EOR activity of the Ni(OH)_2_-PtCu/C catalyst can be explained by the co-existence of surface Cu and Ni(OH)_2_, which can sufficiently promote the water dissociation and thus formation of OH_ads_ species to oxidize the toxic CO on the neighboring Pt surfaces (Erini et al., 2015; Zhang et al., 2015; Liu et al., 2017). Furthermore, the Ni(OH)_2_-PtCu/C also exhibited a lower onset potential than that of the commercial Pt/C. The decrease in the onset potential probably indicates an enhancement in the kinetics of the EOR and may also hint toward an earlier C-C bond break (Erini et al., 2015). These results validate that the electrooxidation of ethanol on the Ni(OH)_2_-PtCu/C is much easier. Although some of Ni(OH)_2_ on the surface was prone to dissolve in acidic media, the Ni(OH)_2_-PtCu/C showed improved catalytic EOR performance and CO oxidation ability compared to the PtCu/C-HOAc, implying the influence of surface Ni(OH)_2_. Consequently, the cooperation effect of Ni(OH)_2_ and Cu leads to a significant increase in EOR activity and kinetic.

**Figure 6:**
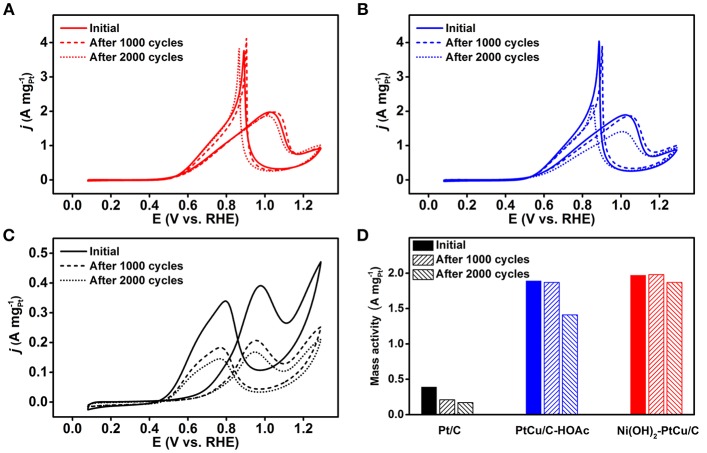
CVs of **(A)** the Ni(OH)_2_-PtCu/C, **(B)** the PtCu/C-HOAc, and **(C)** the commercial Pt/C before and after the durability tests conducted under continuous potential cycling between 0.65 and 1.05 V in Ar-saturated solutions containing 0.1 M HClO_4_ and 1.0 M ethanol with a scan rate of 100 mV s^−1^ at room temperature. **(D)** Comparison of the mass activities of the three different catalysts before and after the durability test.

Chrono-amperometry (CA) curves of Ni(OH)_2_-PtCu/C, PtCu/C-HOAc, and the commercial Pt/C were recorded at 0.67 V for 3,000 s to evaluate the CO tolerance on each catalyst (Figure S3) (Sulaiman et al., 2017). As the reaction proceeded for 3,000 s, the CA curves of Ni(OH)_2_-PtCu/C showed relatively higher and more stable current densities compared to those of the PtCu/C-HOAc and the Pt/C. Because CO intermediate is stable under the continuous operation of EOR on Pt surfaces, this result indicates that the Ni(OH)_2_-PtCu/C has a much higher CO tolerance than the PtCu/C-HOAc and the Pt/C. Long-term durability tests of Ni(OH)_2_-PtCu/C, PtCu/C-HOAc, and the Pt/C for EOR were performed by applying cyclic potential sweeps between 0.65 and 1.05 V at 100 mV s^−1^ in an Ar-saturated solution containing 0.1 M HClO_4_ and 1.0 M ethanol at room temperature. Then, CVs were recorded in a fresh Ar-saturated 0.1 M HClO_4_ and 1.0 M ethanol solution between 0.08 and 1.29 V at 50 mV s^−1^ (Figure 6). After 1,000 cycles, the EOR mass activities of Ni(OH)_2_-PtCu/C and PtCu/C-HOAc were similar to their initial values. In the case of Ni(OH)_2_-PtCu/C, mass activity was slightly increased even after 1,000 cycles. Since the Ni(OH)_2_ can be dissolved out from the Ni(OH)_2_-PtCu/C during EOR cycles under acidic electrolyte, more exposed surface of Pt-Cu and optimized amount of Ni(OH)_2_ can be assumed to show slightly enhanced EOR activity after 1,000 cycles (Cui et al., 2013b). After 2,000 cycles, EOR activities were degraded for all catalysts. Mass activity of the commercial Pt/C catalyst was largely decreased about 57% compared to the initial value. However, both Ni(OH)_2_-PtCu/C and PtCu/C-HOAc catalysts showed 5 and 25% degradation of mass activities to their initial values, respectively. All electrochemical results confirm that Ni(OH)_2_-PtCu/C has improved catalytic activity and durability toward the ethanol electrooxidation.

## Conclusion

We have successfully synthesized the Ni(OH)_2_ decorated Pt-Cu octahedra in one-pot synthesis for EOR electrocatalysts in acidic electrolyte. CO gas was directly introduced in a reaction process as a capping agent on the PtCu(111) facet. Ni(OH)_2_ was naturally deposited on the Pt-Cu alloy octahedra due to the difference of standard reduction potentials of Pt, Cu, and Ni. The presence of Ni(OH)_2_ on the surface of catalysts and Pt-Cu alloy octahedra were confirmed by TEM, EDS, XPS, and XRD analyses. Electrochemical CO stripping experiment showed that enhanced CO tolerance of the Ni(OH)_2_-PtCu/C compared to that of the PtCu/C-HOAc and the commercial Pt/C due to the existence of Ni(OH)_2_ on the surface of Pt-Cu and incorporation of Pt with Cu. Pt-Cu alloy composition showed improved CO tolerance because of the down-shifted Pt *d*-band structure, thereby weakening the CO binding strength on the Pt. In this context, Ni(OH)_2_-PtCu/C and PtCu/C-HOAc showed enhanced catalytic activity and durability toward ethanol electrooxidation in acidic electrolyte compared to the commercial Pt/C.

## Data Availability

All datasets generated for this study are included in the manuscript/Supplementary Files.

## Author Contributions

YH, HK, and S-IC designed experiments, finished the synthesis and characterization analysis of materials, and carried out electrochemical experiment. HL and JK offered valuable suggestions to analysis of materials. YH, HK, and S-IC wrote the manuscript.

### Conflict of Interest Statement

The authors declare that the research was conducted in the absence of any commercial or financial relationships that could be construed as a potential conflict of interest.
