# Supplementary material for: Ni(OH)2 Decorated Pt-Cu Octahedra for Ethanol Electrooxidation Reaction
Source: Front Chem. 2019 Sep 3;7:608. doi: 10.3389/fchem.2019.00608 (PMC6733919; doi:10.3389/fchem.2019.00608)
Supplement: Supplementary file 1 [file Table_1.DOCX]

Supplementary Materials

Ni(OH)_2_ Decorated Pt-Cu Octahedra for Ethanol Electrooxidation Reaction

Youngmin Hong,^†^ Hee Jin Kim,^†^ Hye Jin Lee, Jeonghyeon Kim, and Sang-Il Choi*

Department of Chemistry and Green-Nano Materials Research Center, Kyungpook National University, Daegu 41566, Korea

^†^These authors contributed equally to this work

*** Correspondence:**Prof. Sang-Il Choi
sichoi@knu.ac.kr

# Supplementary Figures

**
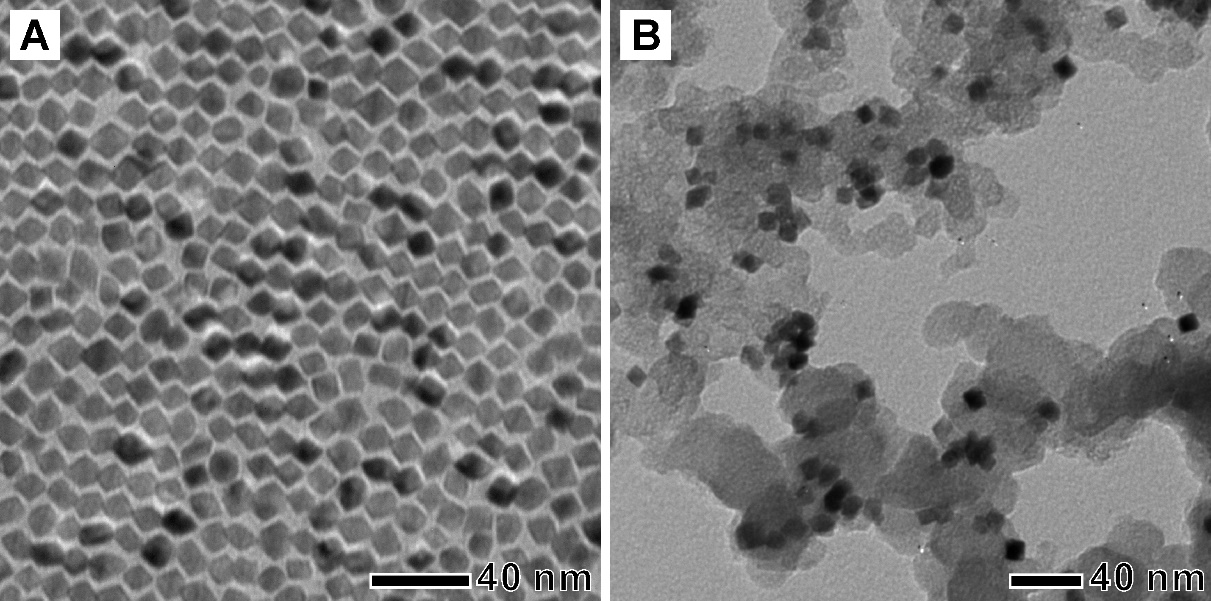
**

**Figure S1.** TEM images of (A) Ni(OH)_2_ decorated Pt-Cu octahedra and (B) carbon supported Ni(OH)_2_-PtCu (Ni(OH)_2_-PtCu/C).


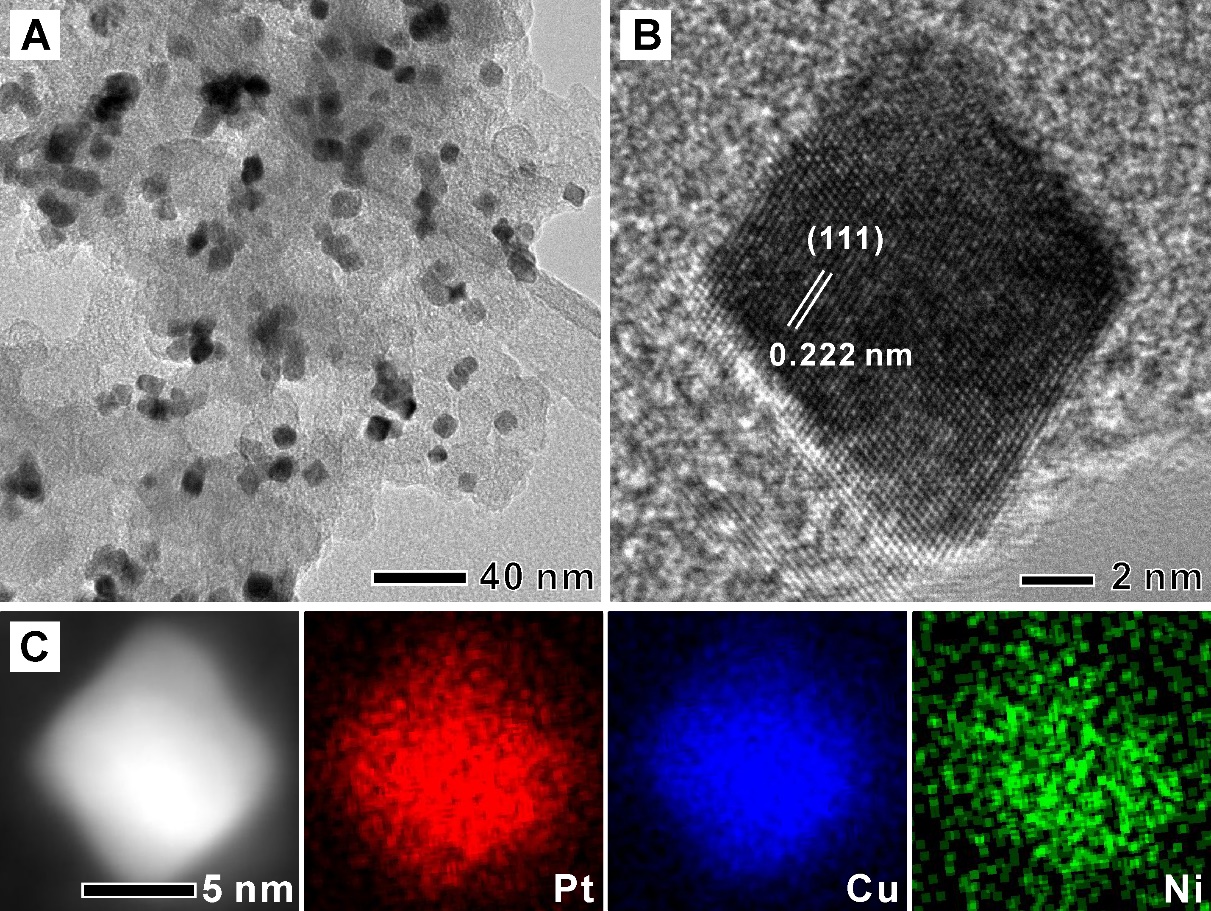


**Figure S2.** (A) TEM, (B) high-resolution TEM, and (C) STEM with corresponding EDS mapping images of Ni(OH)_2_-PtCu after CV clean.


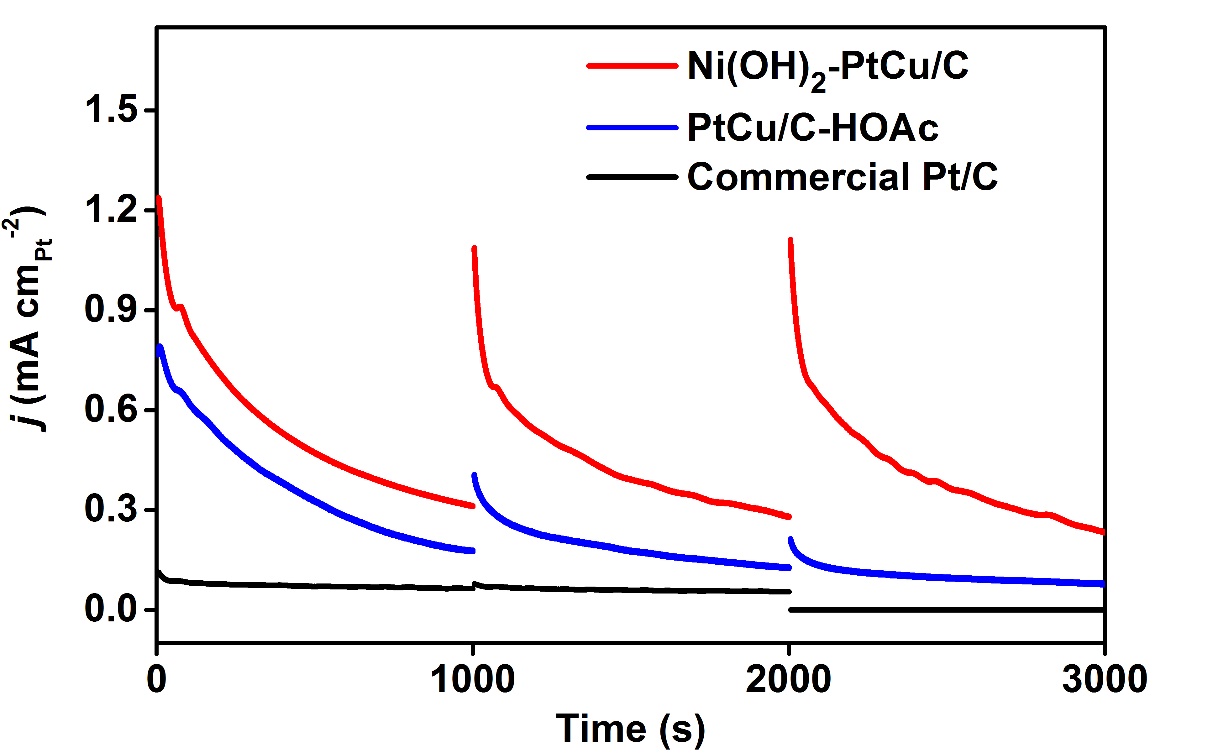


**Figure S3.** Chrono-amperometry curves of the three different catalysts in Ar-saturated 0.1 M HClO_4_ and 1.0 M ethanol at 0.67 V for 3000 s.
